# The electronic Person‐Specific Outcome Measure (ePSOM) in the US population: What matters to individuals the most about their brain health

**DOI:** 10.1002/alz.092530

**Published:** 2025-01-09

**Authors:** Stina Saunders, Ali Jannati, Joyce Rios Gomes‐Osman, Jeff Pobst, William Isaiah Morrow, Sean Tobyne, John Showalter, Alvaro Pascual‐Leone

**Affiliations:** ^1^ Linus Health Europe, Dublin Ireland; ^2^ University of Edinburgh, Edinburgh United Kingdom; ^3^ Harvard Medical School, Boston, MA USA; ^4^ Linus Health, Boston, MA USA; ^5^ University of Miami Miller School of Medicine, Miami, FL USA; ^6^ Hebrew SeniorLife, Boston, MA USA

## Abstract

**Background:**

It is essential that both drug and lifestyle‐based interventions aimed at delaying the functional decline in conditions like Alzheimer’s disease and related dementias (ADRDs) capture change in functioning that incorporates the person’s voice. Such brain health priorities can vary across populations and it is unclear to what degree findings from the ePSOM program in the UK might apply to the US.

**Methods:**

We conducted an online nationwide study to understand what matters to people aged 50 and older about their brain health in the US. The ePSOM US survey (May 2023‐Jan 2024) collected primarily free‐text responses, alongside sociodemographic information, self‐reported neurodegenerative disease diagnosis, and self‐reported confidence in managing personal priorities. We examined [1] the difference in relative frequency of specific words for key sociodemographic groups and comparable UK/US populations, and [2] compared the differences in confidence scores between the diagnosis and non‐diagnosis groups (gender and age controlled) using the Mann–Whitney U test.

**Results:**

A total of 764 participants provided over 11,522 free text responses. Most were female (69%), married (63%), had higher education (64%) and did not report neurodegenerative disease diagnosis (93%); mean age 63.17 (SD = 8.52) in women, 62.99 (SD = 8.30) in men. There were statistically significant differences (p<0.05) in the pattern of responses between sub‐groups: younger/older; male/female; higher/lower education and UK/US populations but not between those with and without a diagnosis. Individuals with a diagnosis rated their confidence significantly lower (median score 20/25, IQR = [17,23]) than those without a diagnosis (median score 24/25, IQR = [22,25]; U = 8908, p<0.05).

**Conclusion:**

Our study demonstrates that there is heterogeneity in the outcomes that matter to individuals about their brain health across sociodemographic groups and UK/US populations. These findings support the notion that emerging treatments in ADRD should be developed with a person‐centred approach that accounts for sociodemographic factors, and that confidence may be an appropriate construct to measure self‐perceived ability. Efforts such as the ePSOM program offer an evidence‐based method to capture and monitor personally meaningful treatment targets.

